# Allogeneic Mesenchymal Stem Cells and Biomaterials: The Perfect Match for Cardiac Repair?

**DOI:** 10.3390/ijms19103236

**Published:** 2018-10-19

**Authors:** Inigo Perez-Estenaga, Felipe Prosper, Beatriz Pelacho

**Affiliations:** 1 Laboratory of Regenerative Medicine, Foundation for Applied Medical Research, 31008 Pamplona, Spain; iperez.27@alumni.unav.es (I.P.-E.); fprosper@unav.es (F.P.); 2 Department of Hematology and Cell Therapy, Clínica Universidad de Navarra, University of Navarra, 31008 Pamplona, Spain; 3 IdiSNA, Navarra Institute for Health Research, 31008 Pamplona, Spain

**Keywords:** myocardial infarction, biomaterials, mesenchymal stem cells, allogeneic patch

## Abstract

Coronary heart disease is the leading cause of death worldwide with huge socio-economic consequences. Cell therapy, and particularly mesenchymal stem cells (MSC), are considered a promising option to treat this disorder, due to their robust trophic and immunomodulatory properties. However, limitations such as their low rate of engraftment and poor survival after administration into the heart have precluded their large-scale clinical use. Nevertheless, the combination of MSC with polymer-made scaffolds or hydrogels has proven to enhance their retention and, therefore, their efficacy. Additionally, their allogeneic use could permit the creation of ready-to-use cell patches able to improve their feasibility and promote their application in clinical settings. In this review, the experimental and clinical results derived from the use of MSC in cardiac pathology, as well as advances in the bioengineering field to improve the potential of therapeutic cells, are extensively discussed. Additionally, the current understanding of the heart response to the allogeneic MSC transplants is addressed.

## 1. Cardiac Diseases: Epidemiology and Etiopathology

Cardiovascular diseases are the leading cause of death worldwide, accounting for 33% of fatalities in people aged more than 35 years. In Europe alone, cardiac events, and mainly myocardial infarction (MI), take the lives of 4 million people per year [1].

MI occurs when blood flow is restricted to a part of the heart, thus causing ischemia in the myocardial muscle. The lack of oxygen in the area induces cardiac tissue necrosis which, in turn, promotes the formation of a scar tissue that impairs heart contractibility, which may lead to heart failure in the mid- to long term. MI can be fatal and survivors exhibit a diminished cardiac function and have reduced life expectancy, even if they were treated promptly. Fortunately, the development of new drugs and the improvement of medical care have decreased the mortality rate and improved the prognosis of MI patients. Despite these advances, the chronic nature of the disease has put great pressure on healthcare systems. In Europe, the annual costs attributable to cardiac ischemic diseases amount to around 200,000 million euros. This figure represents approximately 54% of the total investment in health. Furthermore, these pathologies are responsible for a 24% loss of productivity [2]. The epidemic of coronary heart disease is thus one of the biggest challenges facing the scientific and medical communities.

## 2. Mesenchymal Stem Cells (MSC) for the Treatment of Myocardial Infarction (MI)

In the last two decades, cell therapy has become a new alternative for the treatment of MI. Different types of stem cells, namely bone marrow (BM) and cardiac derived cells, skeletal myoblasts, endothelial progenitor cells, and mesenchymal stem cells (MSCs), have been tested at both the experimental and clinical level (reviewed in [3]). Among these, MSCs have been considered as one of the most promising options for cardiac therapy, due to their paracrine and immunomodulatory properties (reviewed in [4]). Furthermore, MSCs are considered to be immunoprivileged, and are therefore potentially apt to be used as allografts [5,6].

**Figure 1:**
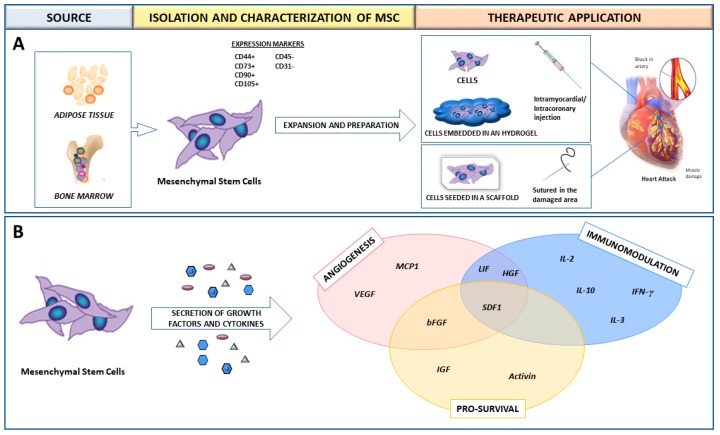
Therapeutic benefit of bone marrow and adipose derived mesenchymal stem cells for cardiac therapy (**A**). Mesenchymal stem cells (MSC) have been applied in combination with biomaterials to enhance their retention in the damaged area. (**B**) MSC exert a paracrine therapeutic action by secreting growth factors and cytokines to the surrounding environment.

MSCs are derived from the stroma of different organs/tissues such as BM or adipose tissue. They are self-renewing and multipotent, and express phenotypically the CD44, CD73, CD90, and CD105 surface markers. Their plastic abilities cover multilineage differentiation towards adipocytes, osteoblasts and chondroblasts [7,8] (Figure 1A).

In the cardiac context, a large number of experimental studies have demonstrated that treating infarcted animals with MSCs exerts a functional benefit, which is mainly due to a series of cytokines and growth factors that the MSCs themselves secrete (reviewed in [4]) (Figure 1B). This paracrine action induces several beneficial effects on the surrounding ischemic environment, such as the promotion of vasculogenesis and angiogenesis, the regulation of extracellular matrix components, and the induction of cell survival [4]. The immunomodulatory abilities of MSCs have also been documented in vitro. MSCs inhibit the activation and proliferation of T and B lymphocytes. Furthermore, when co-cultured with monocytes, MSCs block the differentiation of these into dendritic cells and downregulate antigen-presenting-cell molecules. Finally, MSCs reduce the cytotoxic activity of natural killer (NK) cells [6] and promote maturation of monocytes into anti-inflammatory type M2 macrophages [9].

Importantly, MSCs can be easily obtained from patients, and the associated risk and morbidity are negligible. In sum, the easy availability and the immunomodulatory properties of MSCs have placed them in the focus of both researchers and clinicians, who are keen to use them as an effective therapeutic tool for heart regeneration.

## 3. MSC in Preclinical and Clinical Studies for MI Treatment

The therapeutic potential of MSC for MI has been tested in large preclinical animal models, and cardiac function recovery has been observed, as well as a positive effect on myocardium remodeling subsequent to their administration, with no identifiable safety concerns. Indeed, numerous experiments were previously performed in rodent MI models, which had shown a functional benefit together with reduced scar formation after intramyocardial MSC injection [10,11,12,13]. When the safety of BM-MSC administration was analyzed in a pig model of MI, no differences in mortality, post-injection arrhythmias, cardiac enzyme release, or systemic inflammatory markers, with respect to the control group, were documented [14]. Other experiments in porcine models further confirm the safety of the use of MSC [15,16]. On the other hand, the efficacy of MSC has also been demonstrated in several large animals. In models of chronic ischemic heart disease in dogs and pigs, MSC increased vascularity and improved cardiac function in the former [17], and autologous adipose tissue-derived MSC (ADSC), improved left ventricular (LV) ejection fraction (LVEF), increased wall thickness, and induced vessel formation four weeks after implantation in the latter [18]. Similar effects on LVEF and myocardium remodeling were observed in swine four weeks after ADSC and MSC administration [19]; other porcine MI models further confirmed the usefulness of these cells to improve cardiac function, reduce scar size, and promote adequate remodeling [15,16]. Finally, it is worth mentioning that MSCs have been demonstrated to be superior to dermal fibroblasts when transplanted as a cell-sheet into infarcted rat hearts. Unlike fibroblasts, MSCs significantly improved hemodynamic parameters, increased wall thickness, and augmented left ventricle end-diastolic dimensions. Importantly, the survival rate after transplantation was significantly higher in the animals treated with MSC than in those treated with fibroblasts [20].

The positive preclinical results prompted testing of MSC in the clinical setting. In the first-in-human clinical trial, namely the APOLO study, the safety and effectiveness of the intracoronary administration of autologous ADSC were tested in patients who had acute MI (AMI), with encouraging results. First of all, there were no safety concerns, since no serious adverse effects (SAEs) subsequent to ADSC administration were reported. Furthermore, a 4% increase in LVEF, a reduction of the infarcted area and a significant improvement in perfusion could be observed [21]. The PROMETHEUS trial, focused on chronic disease, obtained additional positive results. Eighteen months after autologous MSC administration, an increase of 10% and 25% in LVEF and LV stroke volume, respectively, could be seen in patients with chronic coronary cardiomyopathy. At that time, the scar tissue mass had been reduced by 8%, and an additional concomitant increase of viable tissue was documented [22].

## 4. Allogeneic Transplant Model

If a cell therapy-based treatment of MI is planned, allogeneic MSC are undoubtedly more suitable than autologous ones. Firstly, in the autologous transplant model, precious time is lost in the in vitro isolation, expansion, and validation of the cells. Secondly, several studies have demonstrated that aging and risk factors for coronary artery disease negatively influence stem cell function, thus limiting their therapeutic potential [23]. Finally, inter-individual variability will also influence success, which will be conditioned by the quality and effectiveness of each patient´s cells. However, provided that a well identified ready-to-use stock of MSC is available at the hospital, the allogeneic model guarantees that the cells to be used will be appropriately characterized following all the GMP (Good Manufacturing Practices)-requirements. Furthermore, these MSC will be fully functional, since they originate from young healthy donors, and will be able to be stored and kept ready to use off-the-shelf. No less importantly, widespread application of the allogeneic model could promote the use of MSC-based therapies, due to the lower costs associated with the logistics, as compared with those linked to the autologous cell procedures (Table 1).

These arguments prompted the design of two clinical trials, namely POSEIDON and PROCHYMAL, intended to assess the safety and efficacy of allogeneic MSC. The first was a phase I–II early-stage study. Alloantigens against the donor were detected in 3.7% of patients only, i.e., no major alloreactivity was found, and the incidence of SAEs was low. Interestingly, an improvement of cardiac function with an increase of systolic volumes and a reduction of the infarct size was documented [24]. Nevertheless, despite these encouraging results, larger studies including a placebo group are required. In the phase-I PROCHYMAL trial, allogeneic BM-MSC were intravenously infused in patients who had experienced an AMI event, which led to an increase of LVEF at three months of 5.9%, higher than that experienced by patients who did not receive cells but placebo instead, i.e., 4.4%. Furthermore, the effect was maintained over six months. As in the POSEIDON trial, there were no safety concerns, since no major SAEs were documented in the treatment group [25]. In any case, there are still a series of concerns regarding this therapy which have to be addressed. PROCHYMAL II is an ongoing phase-II trial which includes larger cohorts of patients, and therefore, should contribute to confirm the safety and efficacy of allogeneic MSC in cardiac repair with certainty, and thus, to pave the way to the incorporation of these procedures in clinical routine.

## 5. Allorecognition of MSC by the Host Immune Cells

The degree of allorecognition that occurs between the host and the allogeneic MSC when these are infused or transplanted into an organism is currently a matter of debate. Several studies have proven that, in the resting state, MSC express no MHC II and low MHC I levels. MSC do not express co-stimulatory molecules, such as CD40, CD80, or CD86, either. Since these are required for effector T-cell recognition, MSC remain “invisible” to the host immune cells [5,26]. However, several preclinical studies still found some degree of immune activation and response by the host subsequent to MSC infusion. In fact, Zangi et al. observed that MSC could not fully evade the host’s immune system in spite of their immunoprivileged status. In their studies, intraperitoneally injected MSC lasted notably longer in immunocompetent mice than in immunodeficient ones (120 vs. 40 days, respectively). Control allogeneic fibroblasts only survived 20 days after infusion into immunocompetent mice. Thus, MSC showed partial immunoprivilege. Accordingly, syngeneic MSC, in turn, lasted longer than allogeneic MSC after intravenous administration into immunocompetent mice (40 vs. 20 days, respectively) [27]. Another study was designed with rats to test the hypothesis that the activation state of MSC would influence the initiation of the immune response. For this purpose, MSC were differentiated into myogenic cells prior to their administration into the host organism to analyze their effects. An increase in the expression of immune antigens, such as MHC-Ia, MHC-II, and CD86, was found in the differentiated cells. In vivo, the undifferentiated cells lasted longer than the differentiated ones at the injection site. Furthermore, the alloantibodies detected in the recipients’ blood had been raised against differentiated allogeneic MSC only [28]. Accordingly, another study recently found that, after being placed in the infarcted epicardium of rats, allogeneic and syngeneic MSC sheets engrafted and survived in a similar way during the early phase, i.e., three and 10 days after implantation, though not in a later phase, i.e., at 28 days, at which time cell survival was considerably more reduced in the epicardially placed allogeneic MSC implants (0.2% vs. 8.9% with syngeneic cells). In addition, local immunological responses induced by these could be observed. The rate of CD4+ T cells that had accumulated on the MSC sheet at days three and 10 was increased in the allogeneic MSC group when compared with that observed in the autologous MSC group, and interleukin-6 (IL-6), which is a pro-inflammatory cytokine reported to play a role in allograft rejection, was upregulated in the allogeneic MSC group, while its expression remained unchanged in the rats treated with syngeneic cells. Despite this, the percentages of CD8+ T cells or CD68+ macrophages accumulated in the MSC sheets were not significantly different between the allogeneic MSC and syngeneic MSC groups. In any case, the beneficial effect on cardiac function recovery and myocardium repair was similar in the allogeneic and syngeneic groups [29]. These findings are in agreement with those obtained in another MI model in rats, since the efficacy of allogeneic or autologous MSC sheets was also comparable because both groups experienced an equal improvement in cardiac function. Furthermore, in this model, the degrees of cell engraftment and immune cell infiltration did not differ between treatments either [30].

At this stage, the information provided by a series of reliable studies suggests that host allorecognition of the exogenous MSC does indeed occur, thus influencing survival of these at the implantation site. Nevertheless, even though a low/mild immune response seems to exist, this would not restrict the therapeutic effect exerted by the implanted MSC. More extensive research at both preclinical and clinical levels should be performed on this specific issue to better understand the immunoregulatory actions of MSC.

## 6. Biomaterials as Co-Adjuvants for MSC Therapy in Cardiac Regeneration

Despite the proven therapeutic potential of MSC, poor engraftment and survival of the transplanted cells within the ischemic myocardium remains an important limitation when it comes to achieving a robust therapeutic effect. Aside from the delivery mode, dose, and time of infusion, several other causes, namely mechanical stress caused by heart contractility, cell apoptosis caused by anoikis, i.e., lack of cell adhesion, and endogenous environmental factors such as hypoxia or host immune response, influence the retention and viability of the infused cells (reviewed in [31]). In order to overcome these drawbacks, some approaches have been developed to increase the engraftment and survival of the cells, which include cell preconditioning by heat-shock, hypoxia, and/or cytokine pre-treatments. Furthermore, the genetic modification of MSC to overexpress pro-survival factors has also been proven to be beneficial [31,32,33]. Notably, the use of biomaterials for improving cell retention and survival has been thoroughly tested in MI animal models and will be discussed next.

A wide range of biomaterials of both natural and synthetic origin have been assayed for cardiac regeneration therapy, either alone or together with cells. Biomaterial features such as composition, elasticity, porosity, viscosity, and micro- and nano-structure largely influence biocompatibility with the host organism. Overall, biomaterials have been applied to the heart either as scaffolds/patches or as injectable suspensions/hydrogels (reviewed in [34]). Natural compounds such as collagen, chitosan, alginate, fibrin, or synthetic materials including polycaprolactone (CPL) or polyglycolic acid (PGA) and polylactic acid (PLA) derivatives, have been used to develop patches and hydrogels. Combinations of natural compounds and synthetic materials have also been developed in order to optimize the mechanical properties of the scaffolds and increase cell adhesion (reviewed in [35]) (Table 2).

## 7. Use of Hydrogels for Cardiac Application

Polymers such alginate, fibrin, or combinations of both have been the most commonly used materials, owing to their gelation properties for percutaneous delivery. Hydrogels alone can provide mechanical support for the infarcted heart, and more interestingly, are able to carry cells to the damaged myocardium. A significant improvement in MSC retention and viability when these are injected in combination with hydrogels has been widely documented (reviewed in [36]). Accordingly, in a rat MI model, intramyocardially delivered BM-MSC survived longer when administered with a fibrin glue hydrogel than when administered alone. As a consequence, cardiac function improved, and recovery correlated with a reduction in the scar size [37]. Interestingly, collagen hydrogels have also been assayed to treat MI. In fact, collagen was found to be superior to fibrin as a cell carrier in another rat model. Even though both polymers increased cardiac ADSC retention, cell survival was higher with collagen [38]. In a slightly different approach, Yu et al. modified alginate microspheres in order to permit MSC encapsulation. The subsequent injection into the damaged myocardium of immunocompetent rats rendered positive results regarding the cell survival rate [39]. Corroborating the efficacy of this strategy, greater retention and therapeutic effect of BM-MSC was also shown when subcutaneously injected in a rat model, after their previous encapsulation in alginate [40].

The promising experimental findings observed with this biomaterial prompted researchers to test their clinical reliability. For this reason, acellular alginate was tested in the phase-I PRESERVATION-I (Prevention of Remodelling of the Ventricle and Congestive Heart Failure After Acute Myocardial Infarction) trial with encouraging results, since these confirmed the safety and feasibility associated with its use. A clinical trial combining alginate with stem cells is currently ongoing [41].

## 8. Cardiac Patches and Cellularized Scaffolds

In the case of scaffolds, collagen has been extensively used, due to its high biocompatibility, effective cell adhesion, and low immunogenicity, although other natural or synthetic polymers such alginate, gelatin, decellularized bovine pericardium, fibrin, or polycaprolactone (PCL), have also been tested to produce cardiac patches [35,42]. In one of the first experiments carried out with cardiac scaffolds, an improvement in cardiac function was shown after implantation of a combinational patch of collagen type I, Matrigel^®^, and rat skeletal muscle cells on rat infarcted hearts [43]. Following a similar approach in another rat model, MSCs were embedded into a collagen-I matrix to form a cardiac patch, which was subsequently sutured to the infarcted heart. Greater engraftment of the cells in the infarct zone could be observed at one week. Interestingly, a significant improvement in cardiac function and anterior wall thickening was also documented later than four weeks after matrix implantation, in spite of the fact that cells had not been detected at 4 weeks, thus suggesting that long-term cell engraftment or survival is not required for MSC to exert therapeutic effects [44]. On the other hand, an engineered ultra-thin collagen type-1 scaffold was seeded with autologous ADSC and subsequently used in a MI porcine model with interesting results. ADSC engraftment was much greater when they were injected in combination with the scaffold support. Furthermore, the animals treated with the cellularized patch showed a significant, correlated increase in LVEF when compared to the control groups, i.e., animals intramyocardially administered either the acellularized patch or the ADSC alone. Finally, positive remodeling of the myocardium and enhanced vascularization were also demonstrated [45]. A different approach consisted of creating patches with decellularized rabbit pericardium, which were later seeded with ADSC and finally used in a rat MI experimental model. Positive results regarding survival and vascularization of the ischemic tissue could be observed [46]. Fibrin has also been widely tested in the development of cardiac scaffolds with different cell types. It is worth mentioning that the functional effect of delivering endothelial and smooth muscle cells combined with a fibrin three-dimensional (3D)-porous scaffold was examined in a porcine MI experimental model. Imaging techniques showed increased engraftment of the transplanted cells and significant LV functional improvement at one and four weeks post-treatment [47].

In the field of synthetic polymers, a series of experiments performed with BM mononuclear cells (BMMC) seeded into biodegradable poly-glycolide-*co*-caprolactone (PGCL) scaffolds showed that this combination led to a progressive reduction of LV dilatation, i.e., to preservation of LV function [48]. With the aim of finding the most effective synthetic polymer for generating cellularized cardiac patches, the ability of caprolactone-*co*-l-lactide (PCLA) to induce cellularization with MSC was compared with that of PGA and gelatin in a rat heart damage experimental model. Although seeded MSC survived by at least eight weeks after patch implantation in all groups, it is noteworthy that a greater degree of cellularization was observed in the cardiac patches when PCLA scaffolds were used [49]. Silk has also been explored as a candidate material for carrying MSC to the damaged heart. In a recent study in rat MI model, cellulose nanofibers modified with chitosan/silk fibroin multilayers was tested, showing a higher cell viability, improved LVEF, and a reduced adverse ventricular remodeling in the treated hearts [50]. Interestingly, self-assembling silk hydrogels have been designed as support for MSC where controllable gel-kinetics could be modified to achieve uniform cell distribution, viability, and space conformity [51].

The positive preclinical results obtained using different biomaterials and cell types invited researchers to test whether these experimental procedures could be translated to the clinical setting. With this aim, the phase I MAGNUM clinical trial was designed with the purpose to compare the effects exerted by BMMC-seeded cellularized collagen matrices with those exerted by cells alone in patients presenting LV post-ischemic myocardial scars. Results were promising in that no treatment-related SAEs were reported during the follow-up period and that heart functionality and mechanical parameters improved significantly in the patients who were administered cellurarized patches. In other words, clinically, this procedure seems to be safe, feasible, and effective [52].

## 9. Acknowledging the Importance of the Scaffold Substrate in the MSC Cytokine Secretion Profile

The undoubted trophic effect induced by MSC, in addition to the well-known influence exerted by tissue/matrix microenvironment on cell behavior led to the design of studies aimed to unveil the scaffold substrate-dependent regulation mechanisms of the pattern of cytokine secretion. In a pioneering study, MSCs were seeded in a TopoWell^®^, i.e., a multiwell plate enclosing 76 unique bioactive surface topographies, in order to analyze subsequently the cell shape and cytokine secretion profile in each separate well. Interestingly, the surface topography did influence cell shape and secretion of pro-angiogenic and anti-apoptotic factors like stromal-derived factor-1α (SDF-1α) and hepatocyte growth factor (HGF) [53]. An analogous strategy was followed to analyze if different microenvironments, ranging from nanoporous hydrogels to macroporous scaffolds, could influence the cytokine secretion pattern of the MSC in three-dimensional cultures. For such purposes, the secretomes of the cells cultured in plastic plates, encapsulated in alginate hydrogels, or seeded in scaffolds were compared. Cells cultured in scaffolds produced several-fold higher concentrations of almost all the 70 cytokines whose levels were measured, when compared with those obtained under the other experimental conditions. The scaffold-seeded cells showed a particularly significant increase in the expression of a series of well-known pro-survival cytokines, namely vascular endothelial growth factor (VEGF), HGF, insulin-like growth factor (IGF,) basic fibroblast growth factor (FGF2), and leukaemia inhibitory factor (LIF) [54]. In a similar manner, a recent experiment employing 3D scaffolds that allowed controlling different parameters such as stiffness, stress relaxation, and adhesion ligand density of the substrate was performed to test if these stimuli affect the gene expression profile of the MSC cultured on it. Indeed, dramatic changes in the MSCs gene expression patterns were observed depending on their specific combinations [55]. These evidences supports the notion that the microenvironment does modulate MSC trophic behavior, and that scaffold composition and topography are able to significantly enhance cytokine and growth factor secretion by MSC. Thus, the ability to modulate the MSC secretome by modifying the scaffold properties highlights the importance of biomaterials for boosting the effects of the MSC-based cell therapy on cardiac regeneration.

## 10. Biomaterials Functionalization with Growth Factors

Since the main therapeutic effects of MSC are due to the release of therapeutic proteins and factors, hydrogels and scaffolds have also been functionalized with such key factors in order to increase their therapeutic potential. Several of these experimental constructs are worth mentioning. A hydrogel with the ability to sequentially release IGF-1 and HGF when injected into the myocardium achieved a therapeutic effect in a rat model of MI [56]. An alginate-based injectable hydrogel that gradually delivered VEGF and platelet-derived growth factor-BB (PDGF-BB) at different rates to accomplish a more consistent effect was engineered [57]. Additionally, a myocardial patch made of collagen membranes loaded with collagen-binding VEGF was generated to be used in a rabbit MI model. Its implantation led to an increase in cardiac function, vasculogenesis, and patch cellularization [58]. Epicardial patches containing follistatin-like 1 (Fstl1), which is a well-known cardiomyogenic factor, were designed as well. Two weeks after their implantation in hearts of myocardial infarcted swine, an increase in cardiomyocyte proliferation, and therefore a regeneration of the cardiac muscle and function, were observed in comparison with animals not receiving such treatment [59]. Experiments have also been performed using a scaffold containing a soft core of gelfoam functionalized with BM-MSC and stromal derived factor-1 (SDF1) and coated with an outer layer of CPL in order to add mechanical strength. Its use in a rat MI model yielded positive results in that cardiac function was recovered, and adequate remodeling and increased vascularization were documented [60].

Finally, another combinational approach has recently been reported. Both VEGF and PDGF were immobilized in MSC-seeded scaffolds [61]. This biomaterial exhibited an enhanced mechanical strength in vitro while maintaining a porous ultrastructure. In addition, the constant release of cytokines promoted the proliferation of the MSC. In rats with LV aneurysm post-MI treated with these VEGF/PDGF-immobilized and MSC-seeded scaffolds cardiac function, cell infiltration and tissue revascularization improved four weeks after their implantation, when compared with rats that had been treated with cytokine-free patches or cell-free patches.

Altogether, these studies highlight the clinical potential of combinational strategies and pave the way for further attempts to boost the natural therapeutic effects of MSC with cytokines and growth factors, to subsequently “ship” these agents into scaffold or hydrogel “carriers” to be implanted in the damaged area of the myocardium.

## 11. Future Perspective: Allogeneic MSC-Seeded and Cytokine-Immobilized Patches for Cardiac Regeneration

**Figure 2:**
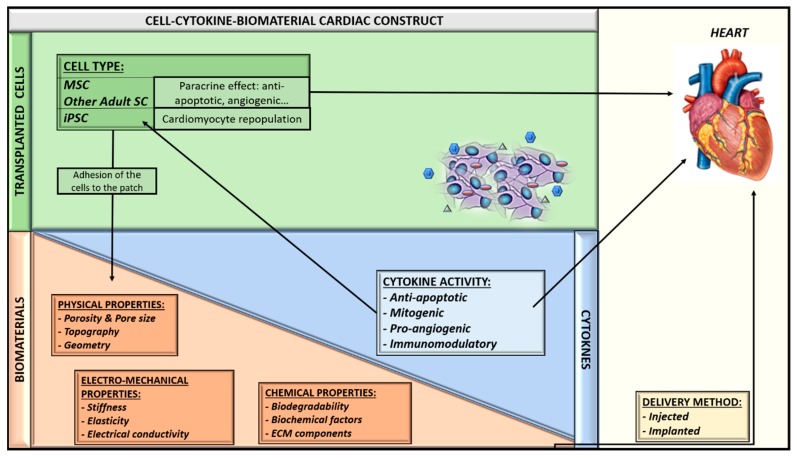
Therapeutic “off-the-shelf” myocardial patches containing stem cells and derived cells and cytokines. **MSC:** Mesenchymal Stem Cells; **SC:** Stem Cells; **iPSC**: induced Pluripotent Stem Cells; **ECM**: Extracellular Matrix.

The aforementioned preclinical models and clinical trials allow us to envision a near future scenario in which cardiac ischemic diseases will be treated with combinations of biomaterials, pharmacological compounds and stem cells. These agents will have been previously manufactured, seeded, and stored “off-the-shelf” in medical centers, thus enabling doctors to use them in an acute phase of the disease with the certainty of having a standardized and validated product available. Nevertheless, before this goal is achieved, many steps must still be taken. Firstly, regarding MSC, the allogeneic transplant setting and the possible reaction of the host immune system to exogenous allogeneic cells need to be better understood. The ongoing PROCHYMAL II clinical trial, as well as others about to be initiated, will surely provide precious information on such topics. Additionally, improvements in biomaterial tissue engineering techniques will allow the creation of scaffolds complying with the ideal biomechanical, biological, and biochemical properties, which are able to deliver cells and therapeutic cytokines at the precise locations and rates required for the appropriate heart regeneration (Figure 2). Moreover, the use of other types of stem cells such as induced pluripotent stem cells (iPSC), which hold the ability to differentiate into cardiac cells, could not only permit better remodeling of the damaged myocardium, but also could lead to the re-population of the cardiac muscle with functional cardiomyocytes. These, in turn, will help to regenerate the damaged heart tissue.

In sum, in the coming years, we are going to address some exciting challenges in the cardiac regeneration therapy field. Multidisciplinary approaches will be combined, and they will surely play a central role in the achievement of the main objective, namely the recovery of cardiac function after myocardial infarction.

## Figures and Tables

**Table 1:** Pros and cons concerning allogeneic and autologous cell transplant.

	ALLOGENEIC MSC	AUTOLOGOUS MSC
*PROS*	Cell availability (“ready-to-use” format) Right trophic properties Lower costs in logistics	Perfect immune match Same MHC haplotype
*CONS*	Low/mild immune response Shorter survival time in the implant area	Cells available at medium term Dysfunctional cells in sick/old patients Higher costs in logistics

**Table 2:** Most commonly used biomaterials in cardiac regeneration therapy.

		Formula	Biodegradability	Stiffness	*E* [kPa]	Cell Adhesion	Other Properties
**NATURAL POLYMERS**	**Gelatin**	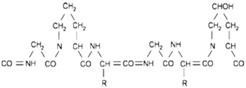	+++	−	0.1−30	+++	Soft, fragile and elastic
	**Collagen**	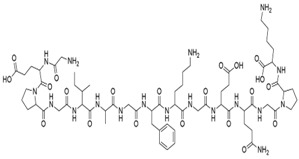	+++	+	0.1−50	+++	Easily cross-linkeable to add strength Natural polymer Remains soluble at low pH and temperature Forms fibers
	**Chitosan**	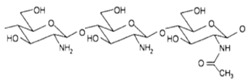	++	+	0.1−50	+	Easy to alter degradation rate Lack of binding sites
	**Fibrin**	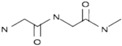	+++	+	0.1−20	++	Porosity and stiffness depend on composition Forms nets of fibers
	**Alginate**	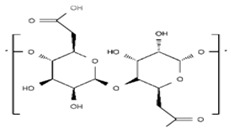	+++	−	0.1−50	++	Large pore size (50–200 µm) Pore size modifiable controlling freezing regime Ideal for hydrogels due to its viscosity
**SYNTHETIC POLYMERS**	**PCL**	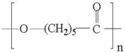	−	+++	>100	−	Easy to modify pore size and structure Highly hydrophobic
	**PGA**	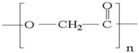	++	++	Depends on composition	+	Lack of structural stability Crosslinkeable
	**PLA**	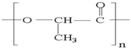	+	++	Depends on composition	+	Variable degradation rate (depending on composition)
	**PLGA**	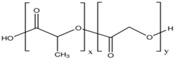	++	+	Depends on composition	+	Variable degradation rate (depending on composition)

(−): None, (+): Low, (++): Medium, (+++): High, (*E*): Young´s Modulus.

## Abbreviations

ADSC	Adipose Derived Mesenchymal Stem Cells
BMMC	Bone Marrow Mononuclear Cell
BM-MSC	Bone-Marrow Derived Mesenchymal Stem Cells
FGF2	Basic Fibroblast Growth Factor
FS	Fractional Shortening
Fstl1	Follistatin-Like 1
GMP	Good Manufacturing Practices
HGF	Hepatocyte Growth Factor
IGF	Insulin-Like Growth Factor
IL-6	Interleukin-6
iPSC	Induced Pluripotent Stem Cells
LIF	Leukaemia Inhibitory Factor
LV	Left Ventricle
MHC	Mayor Histocompatibility Complex
MI	Myocardial Infarction
MSC	Mesenchymal Stem Cells
NK	Natural Killer Cell
PCL	Polycaprolactone
PDGF	Platelet-Derived Growth Factor
PGA	Polyglycolic Acid
PGCL	Poly-Glycolide-*co*-Caprolactone
PLA	Polylactic Acid
SAE	Secondary Adverse Effects
SC	Stem Cell
VEGF	Vascular Endothelial Growth Factor
